# Infectious dengue vesicles derived from CD61+ cells in acute patient plasma exhibited a diaphanous appearance

**DOI:** 10.1038/srep17990

**Published:** 2015-12-11

**Authors:** Alan Yi-Hui Hsu, Shang-Rung Wu, Jih-Jin Tsai, Po-Lin Chen, Ya-Ping Chen, Tsai-Yun Chen, Yu-Chih Lo, Tzu-Chuan Ho, Meed Lee, Min-Ting Chen, Yen-Chi Chiu, Guey Chuen Perng

**Affiliations:** 1 Department of Microbiology and Immunology, Tainan, Taiwan; 2 Institute of Basic Medical Sciences, College of Medicine, Tainan, Taiwan; 3 Center of Infectious Disease and Signaling Research, Tainan, Taiwan; 4 Institute of Oral Medicine, National Cheng Kung University, Tainan, Taiwan; 5 Tropical Medicine Center, Kaohsiung, Taiwan; 6 Division of Infectious Diseases, Department of Internal Medicine, Kaohsiung Medical University Hospital, Kaohsiung, Taiwan; 7 Department of Internal Medicine, School of Medicine, Kaohsiung, Taiwan; 8 Center for Dengue Fever Control and Research, Kaohsiung Medical University, Kaohsiung, Taiwan; 9 Department of Internal Medicine, National Cheng Kung University, Tainan, Taiwan; 10 Institute of Bioinformatics and Biosignal Transduction, College of Bioscience and Biotechnology, National Cheng Kung University, Tainan, Taiwan

## Abstract

The levels of neutralizing antibody to a pathogen are an effective indicator to predict efficacy of a vaccine in trial. And yet not all the trial vaccines are in line with the theory. Using dengue virus (DENV) to investigate the viral morphology affecting the predictive value, we evaluated the viral morphology in acute dengue plasma compared to that of Vero cells derived DENV. The virions in plasma were infectious and heterogeneous in shape with a “sunny-side up egg” appearance, viral RNA was enclosed with CD61+ cell-derived membrane interspersed by the viral envelope protein, defined as dengue vesicles. The unique viral features were also observed from *ex vivo* infected human bone marrow. Dengue vesicles were less efficiently neutralized by convalescent patient serum, compared to virions produced from Vero cells. Our results exhibit a reason why potencies of protective immunity fail *in vivo* and significantly impact dengue vaccine and drug development.

Sufficient immune response is a critical element in the success of a vaccine trial, especially the indicator index–which is robust antibody production. Hence, the levels of neutralizing antibody become one of the surrogate values for predicting vaccine efficacy in clinical trials. However, not all the vaccines in clinical trials are in line with the key performance index. One example would be the dengue vaccine. The efficacy of current dengue vaccine in clinical trials does not meet the protective expectations^1^,^2^, in spite of high antibody titers observed in vaccine recipients. As such, antibody titers defined in *in vitro* neutralization assays with *in vitro* propagated virus preparations have failed to correlate with protective immunity *in vivo*^3^,^4^,^5^.

An effective vaccine against dengue is urgently needed since the escalating dissemination of the mosquito-borne virus has been tabulated more than 100 countries globally^6^ with an estimated number of 400 million new infections and 25,000 deaths annually^6^. The disease manifests a spectrum of symptoms, ranging from asymptomatic, mild dengue fever, to severe dengue hemorrhagic fever and death. Dengue is one of the most challenging diseases to diagnose and treat, because initial symptoms are similar to common febrile illnesses thus leading patients to seek help at very late times during infection^7^. Currently, there are no anti-viral modalities or approved vaccines to treat or prevent dengue, while palliative care with close monitoring is the current practice.

Dengue is caused by dengue viruses (DENV), consisting of four serologically distinct RNA viruses (DENV1 to DENV4) in the family Flaviviridae, genus Flavivirus^8^. The viral genome is a single-stranded, positive-sense RNA that shares the property and function of mRNA allowing the naked RNA to be infectious^8^. *In vitro* the viral RNA encodes a single large protein that is proteolytically cleaved into multiple individual viral proteins, including three structural proteins–capsid (C), membrane (M/prM) and envelope (E)–and seven nonstructural proteins (NSs)^8^.

Virus found in the blood of dengue patients can be easily propagated in cell culture. But attempts to visualize the morphology of the virus in preparations of patient plasma/serum concentrates by electron microscopy (EM) have, so far failed to reveal the presence of classical viral particles^9^. Furthermore, the scarcity of patient antibody against the capsid and the E protein domain III in dengue patients have been documented^10^,^11^. This line of evidence suggests that the corresponding biological properties of DENV circulating in dengue patient are unique and deserve investigation.

## Results

### Sunny-side-up appearance of dengue virions in acute dengue plasma which did not contain DENV capsid protein

**Figure 1:**
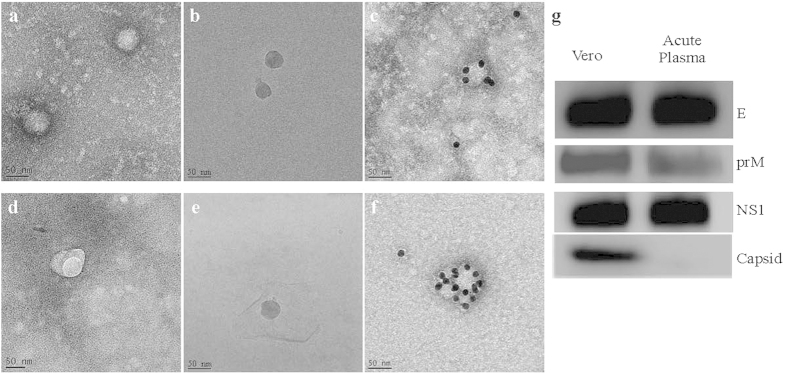
*In vivo* DENV exhibited a sunny-side up egg appearance that was distinctly different from dengue virions from Vero cells. Dengue virions were observed by negative stain EM (**a**) Vero cells, and (**d**) acute plasma) and cryo-EM ((**b**) Vero Cells, and (**e**), acute plasma). A distinct “sunny-side up egg” morphology exhibiting dense-circular structures surrounded by an irregular membrane was seen in acute plasma. Immuno-EM with anti-DENV envelope confirmed that both virions (**c**) Vero cells) and (**f**) acute plasma) were DENV particles. (**g**) Biological properties of dengue vesicles. The lack of capsid protein in the acute plasma derived DENV, while presence of the DENV envelope (E), precursor membrane (prM), and non-structural protein 1 (NS1) were comparable with Vero derived DENV.

Infectious dengue virus was found to reside in micro-particles through fractionation and sequential centrifugation of acute dengue plasma (Extended Data Fig. 1a,b). We therefore investigated the viral entity in the circulation of acute dengue patients. Highly viremic plasma samples were obtained, alone or pooled, concentrated, and directly subjected to EM and cryo-EM analysis. Under the EM, comparing to DENV derived from infected Vero cells (Fig. 1a), the viral morphology from patient acute plasma (Fig. 1d) appeared unique. DENV from acute patient plasma had an extra irregularly shaped membrane surrounding a distinct circular vesicle (Fig. 1d and Extended Data Fig. 2). The appearance was similar to a sunny-side up egg morphology compared to the typical viral particles from Vero cells (Fig. 1a). The unique morphological appearance was further depicted by cryoEM, which showed the distinct difference between the circular form of DENV derived from Vero cells (Fig. 1b) compared to the DENV from acute plasma which had mosaic membranes surrounding the viral particles (Fig. 1e). Immuno-EM investigations confirmed, in parallel with classical virions derived from DENV infected Vero cells, the identity of these vesicles were indeed dengue virions (Fig. 1c, f, respectively). This sunny-side up egg viral morphology was thus designated as “dengue vesicles”.

Biological assays revealed that the dengue vesicles from acute dengue plasma did not have detectable levels of DENV capsid protein, while other viral structure proteins Envelope (E) and pre-cursor membrane (prM) levels were comparable to Vero derived DENV, the non-structural protein 1 (NS1) levels were also similar to that produced from classical virions (Fig. 1g).

### Infectious dengue vesicles contained DENV RNA and were infectious while being morphological interchangeable with classical virions

**Figure 2:**
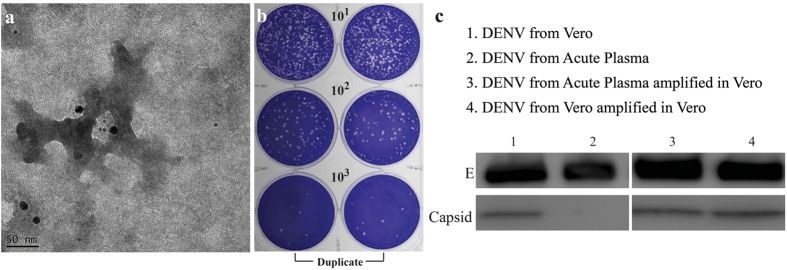
Dengue vesicles contained dengue viral genomic RNA, were highly infectious, and expressed capsid protein when grown in Vero cells. Immuno-EM was performed for dengue vesicles, which were probed with 18 nm gold conjugated with anti-DENV envelope monoclonal antibody and 6 nm gold conjugated with viral RNA hybridized with anti-DENV anti-sense RNA as described in the Methods. (**a**) Dengue vesicles contained dengue viral envelope antigen, appeared as the size 18 nm gold labeling and encapsulating the dengue viral RNA, appeared as the size 6 nm gold labeling. (**b**) Infectious dengue vesicles formed plaques in BHK cells. BHK cells were seeded in 6-well plates, and a duplicate of serial 10-fold dilutions of plasma were added to each well and performed as described in the Methods. The viral plaques were observed after 7 days of incubation. (**c**) Dengue vesicles apparently lacked the capsid protein, but the capsid protein became apparent when these vesicles were used to infect Vero cells suggesting an interchangeable dual life cycle for DENV.

The viral RNA genome inside the dengue vesicle was detected by hybridization with antisense nucleotide to the 3′ end of the viral genome followed by EM imaging, demonstrating that dengue vesicles indeed contained viral RNA (Fig. 2a) and were infectious since it readily formed plaques on indicator BHK cell monolayers (Fig. 2b). Furthermore, the dengue vesicles could regain the capsid content association with the viral particles after a round of infection in Vero cells (Fig. 2c). This result demonstrated differences between *in vivo* and *in vitro* forms of dengue virions which were interchangeable depending on the infected cell and may potentially explain why the antibody response profiles in dengue patients differ from those in mice infected with Vero cell grown DENV^10^,^11^.

### Biological features of dengue vesicles were universal in all dengue serotypes

Investigations with high viral titer acute dengue patient plasma from all DENV serotypes (Extended Data Fig. 3a) revealed that the “sunny side-up egg” appearance (Extended Data Fig. 3b) and the unique biological properties of dengue vesicles in circulation of acute patient plasma were universal for all dengue serotypes (Extended Data Fig. 3c). Thus, we concluded that the *in vivo* dengue virions were different from conventional *in vitro* laboratory grown virions as regards in both morphology and absence/presence of the capsid protein for all serotypes.

### Heterogeneous dengue vesicle sizes were present in patient plasma

**Figure 3:**
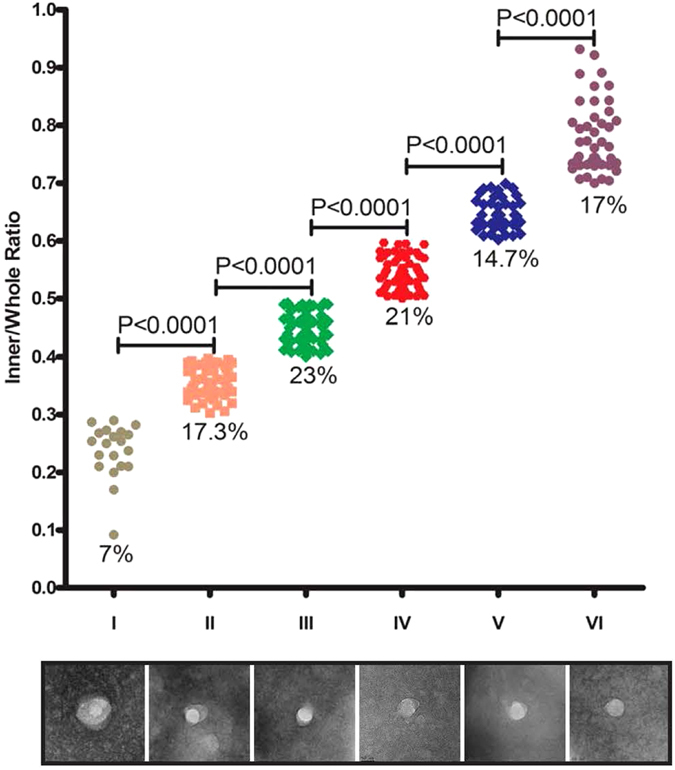
Dengue vesicles were heterogeneous in size. The ratio of the number of pixels within the cyclo-ring inner structure and the whole membrane bound vesicle was measured, and six distinct groups of viral morphologies were categorized. The percentages under each respective population indicate the distribution frequency of dengue vesicles tabulated. Representative images are shown below for each group and in Extended Data Fig. 2.

Various sizes of dengue vesicles were observed and subsequently grouped into six distinct populations based upon the ratio of circumference area of the inner circle (egg-yolk like appearance) to the outer membrane (egg white appearance) (Fig. 3 and Extended Data Fig. 2). The results were in line with previous reports that infectious DENV in acute dengue plasma is heterogeneous in both density and size^9^,^12^.

### Dengue vesicles associated with host CD61 on the membrane suggesting dengue vesicles could be derived from megakaryocyte cells

Systematic human bone marrow studies done in the early 1960 s on dengue patients and recently on rhesus macaque showed that DENV apparently attacks bone marrow cells, especially the megakaryocytic lineage cells^13^,^14^,^15^,^16^,^17^,^18^. Multi-color FACS analysis for hematopoietic stem/progenitor cells and megakaryocytic lineage cells were performed to identify the infected dengue viral NS1 antigen positive cells in the circulation of acute patient blood. Results revealed that DENV NS1 positive cells in acute patient blood were dominantly megakaryocytic lineage cells, where the cell populations were positively correlated to the viral load in the acute patients (Extended Data Fig. 4a), implying viremic DENV was likely derived from these lineages of cells. Immuno-EM investigations demonstrated that one of the host proteins associated with the dengue vesicle membrane (egg white portion) was the megakaryocytic lineage marker CD61 protein (Extended Data Fig. 4b). This result is in orchestra with previous reports of cell surface CD61 positive cells being associated with dengue viral antigens^16^,^18^.

### Dengue vesicles derived from *ex vivo* infected human bone marrow presented the same properties as DENV in acute patient plasma

**Figure 4:**
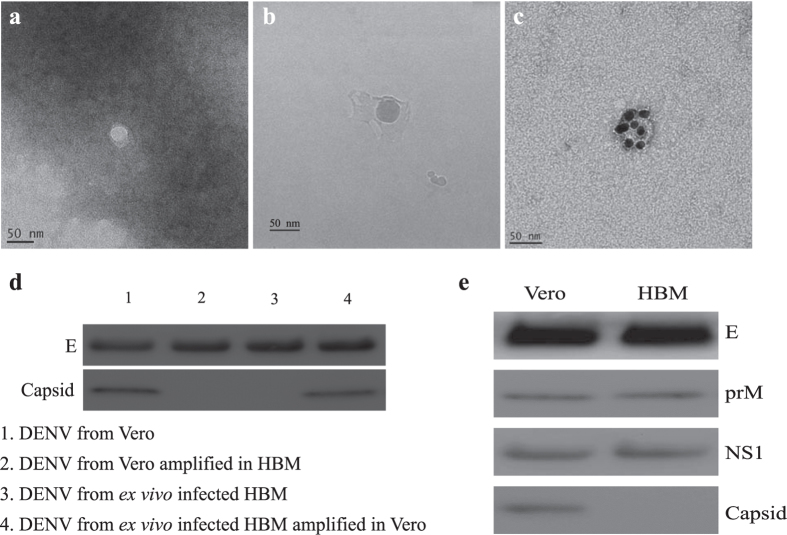
Dengue virus purified from infected human bone marrow demonstrated the same characteristics as virus derived from acute patient plasma. (**a**) DENV from infected human bone marrow were concentrated as described in the Methods and subjected to electron microscopy (EM), cryoEM (**b**) and immunoEM (**c**) confirming the virus particles to be indeed dengue virus. (**d**) Further investigations showed that the dengue vesicles from human bone marrow could also regain the DENV capsid content when amplified in Vero cells, showing the dual life cycle property as seen with DENV from patient serum. **(e)** Protein profiling of virus derived from infected human bone marrow was assayed with Western blot. The results were similar to that of DENV isolated from acute serum, which did not contain capsid but expressed comparable levels of other structure proteins and NS1 levels.

Dengue virions recovered from supernatants of *ex vivo* infected human bone marrow cells presented the same diaphanous morphology and biological properties to dengue vesicles observed in acute patient plasma (Fig. 4) including morphological uniqueness visualized by EM (Fig. 4a), cryoEM (Fig. 4b), and immunoEM (Fig. 4c). Biological assays also showed that the human bone marrow derived DENV did not contain the DENV capsid protein while expressing comparable amounts of other DENV associated proteins (Fig. 4d). Dengue vesicles from infected human bone marrow could also regain capsid content to become classical viral particles when used to infect Vero cells (Fig. 4e). In addition, CD61+ cells purified from bone marrow were highly permissive to DENV infection (Extended Data Fig. 5). We therefore possessed a resource to produce the *in vivo* like dengue vesicles for standard plaque reduction neutralization test (PRNT) to evaluate the quality and efficiency of antibody protective capacity in convalescent dengue serum by comparing against the *in vitro* derived classical virions.

### Viral morphology dictated the neutralizing antibody efficacy in patient convalescent serum

**Figure 5:**
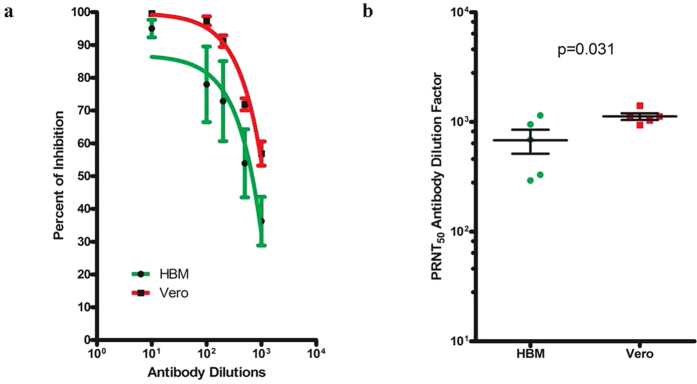
Dengue vesicles were less likely to be neutralized by convalescent dengue serum. PRNT assays were performed in parallel as described in the Methods section. **(a)** Dengue vesicles derived from freshly infected HBM were less likely to be neutralized by the same dengue patient serum in comparison with laboratory dengue virus derived from Vero cells. (**b**) PRNT_50_ was defined as the serum dilution factor that could inhibit 50% of the input virus. The PRNT_50s_ from the samples used in **(a)** were compared and found to be different (p = 0.031).

Cumulative results suggested that dengue vesicles were less likely to be neutralized than Vero-derived virions (Fig. 5a), despite the variation in the capacity and potency of serum neutralizing antibodies to dengue vesicles among individuals (Extended Data Fig. 6). The dilution factor required to reach PRNT50 with dengue vesicles was significantly lower than that of virus from Vero cells (Fig. 5b), suggesting that higher antibody titers were needed to neutralize dengue vesicles.

## Discussion

One of the major obstacles in dengue vaccine development is to find protective parameters that are useful in prediction of efficacy^4^. A consequence due to differences in epitopes recognized by patient sera has been attributed as a potential reason^10^,^11^,^19^ while alternate viral morphology *in vivo* has been suggested to be a factor as well^20^. We report here that all serotypes of dengue virus exist as dengue vesicles *in vivo* and lack the viral capsid protein associated with the highly infectious vesicles, suggesting a likelihood of a dual life cycle and interchangeable form of dengue virus in the natural setting, dependent upon the infected cell properties in the contents of the human bone marrow. This distinct property of DENV may explain why it has been such a difficulty to identify the correlates of protective immunity *in vivo*, since traditionally these assays were performed with virus derived from Vero cells^3^,^4^,^5^. In addition, despite the outcomes of potential dengue vaccines in the phase III clinical trials are available^1^,^2^, the enigma in finding an index for evaluating vaccine efficacy^4^, especially the correlation of neutralizing antibody titers to that of clinical protection^21^, is a problematic issue. The significant difference in DENV properties between *in vivo* and *in vitro* observed in the current report could pave a new concept to explore novel strategies on the alternative dengue vesicles for dengue vaccine development and provide new avenues to evaluate the protective indices, such as the neutralizing antibody titer in dengue vaccine clinical trials.

Interestingly, the concept that DENV may possess complicated life cycles in nature has been proposed before^9^ and two forms of dengue virions, dependent upon the cell type and methods to visualize dengue viral particles, have been noticed in multiple preparations^9^,^22^,^23^,^24^,^25^,^26^,^27^. Regardless of the physical differences, both virions were shown to be infectious^25^. Furthermore, a recent report showed that antibody-dependent enhancement seems to only occur in assays with laboratory-adapted virus and not virus from patient plasma^28^. Therefore, our findings corroborate the presence of the dengue vesicles and may also explain why anti-capsid antibody in dengue patients are rarely detected^29^. However, the current results were solely obtained from acute dengue patients, but it warrants further investigations to address if dengue vesicles are also present in asymptomatic individuals with high viral load and have similar heterogeneous morphology in circulation. Since these findings will advocate that there are two distinct types of dengue virions existing in nature.

Although cells with phagocytic properties have been implicated as the likely targets of initial DENV infection^30^, recently other cell lineages have been shown to be serve as targets of infection^15^,^18^,^31^. The association of host cellular protein CD61 with the dengue vesicles may partially explain the inefficiency of neutralization by convalescent dengue serum. Furthermore, the virus may take advantage to escape from or alter the development of corresponding host cells. The reason dengue vesicles lack the capsid protein while gaining host membranes remains in a shroud. One possibility could result from unique biological and metabolic characteristics of stem/progenitor or megakaryocytic cells which contribute to the degradation of the protein^32^,^33^. Another possibility would be that DENV alters the route of viral maturation pathways in these cells resulting in structural differences in particle contents during infection^34^. As such, what contributes to DENV preferentially targeting stem/progenitor cells and/or megakaryocytic cells and the mechanisms for why the capsid content disappears in *in vivo* setting and attains it in *in vitro* conditions remains to be explored. Delineating the dual life cycle steps between *in vitro* and *in vivo* could lead to more successful vaccine and new drug candidate development and understating of Dengue as a whole.

## Methods

### Patient sample handling

#### Ethics statement for human bone marrow procurement and samples of dengue patients

Bone marrow cells were obtained from the Department of Hematology and Oncology Laboratory of the College of Medicine at National Cheng Kung University. The experiments were conducted following the appropriate approval by the NCKU IRB (Institutional Ethics Committee) with approval protocol # A-ER-102-199. All donors gave written informed consent for the study.

Samples of acute dengue patients were approved by the institutional review board of Kaohsiung Medical University Hospital (KMUH), IRB protocol # 960195 and National Cheng Kung University Hospital (NCKUH) IRB #A-BR-101-156. Enrolled patients were people older than 15 years of age, who visited KMUH and NCKUH and were diagnosed with acute dengue virus infection. Infections were confirmed by the laboratory standards set forth by Taiwan CDC and in accordance with the WHO diagnostic guidelines. To catch and obtain early febrile dengue cases with high viremia in outpatient clinics is highly challenging. We were able to identify 10 cases for the current investigations. In both cases, the samples were collected before or on the third day after fever onset, while the viral loads were confirmed by plaque assay. We observed that viral titers in acute plasma were significantly higher than that of serum (Extended Data Fig. 7). Hence, the viral contents in acute plasma were utilized for the current electronic microscopy (EM) investigations.

### Bone marrow handling procedures

The detailed procedure of handling freshly obtained bone marrows have been described previously^15^. Briefly, the sample from the original biological container, sometimes a syringe or IV bag, was removed. Care was taken to ensure the sample remained sterile. After initial blood smears to ensure cell viability, cells were treated with RBC lysis buffer (QIAGEN #386516) for 10 minutes on a mild shaker, after ensuring full RBC lysis we centrifuged the cells at 300 g for 6 min. Cells were counted and distributed into tubes before they were infected with Vero-derived dengue virus serotype 2, 16681 strain, at MOI = 0.1 for 2 hours with mild mixing every 20 minutes. Then the cells were washed with serum free RPMI by centrifugation and resuspension three times before they were distributed into different tubes and cultured in 2 ml of 10% FBS RPMI (Gibco #11875–093). Virus was harvested at assigned times.

### Dengue virus quantification (qRT-PCR and plaque assay)

Isolation of dengue viral RNA and the quantitative RT-PCR for dengue viral RNA genome was performed as described previously^15^. The universal primer (antisense 5′ GCT CTG TCA CCC AGA ATG GCC AT3′; nucleotide 2192–2170) was used to amplify the viral RNA and could recognize all 4 Dengue virus serotypes^15^. This primer was utilized for our RNA hybridization experiments as described below for the EM detection of DENV RNA in the DENV vesicles.

The viral plaque assays from both acute dengue serum and plasma were performed according to the protocol described previously^35^. Briefly, BHK cells within 10 passages were seeded into 6-well plates, 1 × 10^6^ cells per well, with the addition of 2 ml 5% FBS, DMEM media, and the plates were incubated at 37 °C for 16 to 24 hours. The media was removed gently by aspiration and 400 μL of a 10-fold serial dilution of virus was layered on top. The plates were incubated at 37 °C for 2 hours with shaking of the plate every 15 minutes to insure that the plates did not dry up. After absorption, the infecting media was removed and 3 ml of 1.2% Methyl Cellulose (MC) media (with 2%FBS RPMI and pH = 7.6–7.8) was added per well and incubated at 37 °C for 5 to 7 days. Plates were harvested when plaques were visible to the naked eye. MC media was removed, the plates were washed 3 times with PBS to ensure full removal of the MC media, and stained with the addition of 1% crystal violet solution to each well for 3 hours. Plates were rinsed with tap water and plaques were counted.

### Electronic microscopy

A standard negative stain electron microscopy approach was performed. Briefly, 4 μl of the concentrated viral sample was adsorbed onto glow-discharged carbon-coated grids, stained with 4 μl of 2% uranyl acetate and then air dried. Images were recorded with a JEM1400 transmission electron microscope using an accelerating voltage of 120 kV. The images were recorded directly on a 4 k×4 k Gatan 895 camera with a 14 μm pixel size

### Cryo-electron microscopy

4 μl of the concentrated viral sample were applied onto glow‐discharged copper grids coated with a thin holey carbon film and subsequently plunge‐frozen in liquid ethane using a Gatan CP3 plunging device. The data was recorded under low‐dose conditions (∼10 e/Å^2^ per exposure) in a JEOL2100F with an accelerating voltage of 200 kV. The images were recorded directly on a 3 k×4 k Direct Electron DE-12 camera with a 6 μm pixel size.

### Immuno-Electron microscopy (immuno-EM)

A glow-discharged carbon-coated grid was placed in a humid and low temperature environment. 4  μl of freshly concentrated virus sample was applied onto the grid and then incubated with anti-DENV envelope monoclonal antibody (clone 4G2, CTK B7052 USA) or anti-Human CD61 (AbD serotec MCA2588GA). After incubating for 15 minutes, the sample on the grid was washed multiple times, followed by a similar incubation with 18 nm gold labeled secondary antibodies (ABcam ab105278). After multiple washes the samples were stained with uranyl acetate for 1 minute, excess liquid was removed with filter paper and then air dried. Images were recorded with a JEM1400 transmission electron microscope at 120 kV.

### DENV RNA hybridization and envelope protein double labeling

We pretreated the nickel grid mesh with plasma layering, then directly adding the concentrated and purified patient plasma sample onto the grid followed by surface staining of the Dengue virus particle as aforementioned. Upon fixing the sample using 0.25% glutaraldehyde solution (sigma #G5882), and permeabilizing it with 0.1% triton-X100 (sigma #T7878) to expose the viral RNA. We then probed the dengue virus with the cDNA probe as used in (S. Noisakran 2009), which we conjugated biotin to the 3′of the probe and using anti-biotin-6 nm gold (EMS #25244) to identify the DENV RNA content.

### Measuring viral particle to associated membrane ratio in EM images

Image J (freeware) was used to perform measurements. We standardized and calibrated the scale of pixel length with the scale bar from the raw EM image (analyze/set scale). We gated either the outer border of the viral vesicle or only the inner viral particle and then measured its values for calculations.

### Purification of CD61+ positive cell from human bone marrow

CD61+ cells were isolated via magnetic bead isolation kit (MACS #130–051–101). Briefly, RBC was lysed and the remaining cells were counted. Antibody staining concentration was in line with kit protocols with 20 ul per 1 × 10^7^ cells and with 80 ul per unit. After staining for 30 minutes on ice, cells were washed with washing buffer (MACS #130–091–221) and resuspended in 500 ul washing buffer followed by placing the cells in a pre-rinsed LS column (MACS# 130–042–401) attached to a magnetic holder and steel plate (MACS# 130–090–976). After rinsing the column with washing buffer for 3 times which the flow through contained the CD61− or the antibody unbound cells. We then removed the LS column from the magnetic holder to elute the CD61+ cells which were captured by the antibodies and were thus bound to the magnetic field via the magnetic bead conjugation on the CD61 antibody, after multiple times of elution we could collect the CD61+ cells in the column.

### Viral western blot

A standard protocol was adapted for the Western blot assay. Briefly, the same pfu of virus supernatant was loaded into separate wells of the precast gel (BioRad #456–1084). We then probed the membrane for specific viral proteins E (clone 4G2, CTK B7052 USA), prM (Genetex gtx108092), NS1 (Genetex gtx103346) and Capsid (A gift from Dr. John G Aaskov, Clone 6F3–1).

### Virus purification

A few approaches had been attempted, including ultracentrifugation through gradients, but the outcomes were not satisfactory. We therefore utilized an alternative method to concentrate the virus from plasma. After pooling the plasma virus together, we diluted the viral supernatant with Tris buffer (Ambion #AM9850G) 1:1 in spin columns (amicon ultra 100 kd Millipore #UFC910096) and centrifuged them at 4000 g at 4 °C for 10 to 20 minutes. The procedure was repeated until all of the viral supernatant was used and condensed to 1% of the original volume. The remaining liquid in the column was removed and the membrane was rinsed with 200 ul of Tris buffer. The preparations were stored at −80 °C until used.

### Intracellular detection of dengue NS1 protein in acute patient PBMC

Dengue patient samples were obtained according to patient sample handling protocols mentioned above. PBMC was isolated using Ficoll® Paque Plus (GE17–1440–02, SIGMA) and through subsequent centrifuging. Cells were counted and stained with cell surface markers of defined lineage of cells (BD pharmingen) for stem/progenitor cells and megakaryocyte cells. After staining the cells with the cell surface markers mentioned above on ice for 1 hour, and then washing the cells with washing buffer (0.1% BSA in PBS) we then fixed the cells with 2% paraformaldehyde (P6148, SIGMA-ALDRICH) in PBS and on ice for 30 minutes, followed by permeabilization with 1%saponin (S4521, SIGMA) in PBS for 30 minutes on ice. After thorough washing we then intracellularly stained the cells with dengue NS1 specific antibodies (B7140, CTK Biotech) conjugated with alexa-fluor 647 (Life tech, A-20173) on ice for 1 hour. Cell sub-populations were processed with BD LSRFortessa and analyzed the results using Kaluza 1.3 software from Beckman to identify the NS1 expression in each sub-population.

### Plaque Reduction Neutralization Test (PRNT)

The PRNT was performed with WHO guidelines^35^. Briefly, convalescent dengue serum confirmed with rapid test kit (CTK#R0061C), was incubated at 560 C for 30 minutes to inactivate the complement components. Serum with appointed dilutions was incubated with DENV derived from Vero or from human bone marrow for 1 hour at 37 °C with inverted mixing every 30 minutes. We then preformed dengue virus quantification by plaque assay method as aforementioned. The PRNT50 was calculated with GraphPad Prizm software program (www.graphpad.com).

## Additional Information

**How to cite this article**: Yi-Hui Hsu, A. *et al.* Infectious dengue vesicles derived from CD61+ cells in acute patient plasma exhibited a diaphanous appearance. *Sci. Rep.* **5**, 17990; doi: 10.1038/srep17990 (2015).

## Supplementary Material

Supplementary Information
