# Supplementary material for: Infectious dengue vesicles derived from CD61+ cells in acute patient plasma exhibited a diaphanous appearance
Source: Sci Rep. 2015 Dec 11;5:17990. doi: 10.1038/srep17990 (PMC4675971; doi:10.1038/srep17990)
Supplement: Supplementary Information [file srep17990-s1.pdf]

Supplementary Information

Infectious dengue vesicles derived from CD61+ cells in acute patient plasma exhibited a diaphanous appearance

Alan Yi-Hui Hsu, Shang-Rung Wu, Jih-Jin Tsai, Po-Lin Chen, Ya-Ping Chen, Tsai-Yun Chen , Yu-Chih Lo ,Tzu-Chuan Ho, Meed Lee, Min-Ting Chen, Yen-Chi Chiu[4], and Guey Chuen Perng

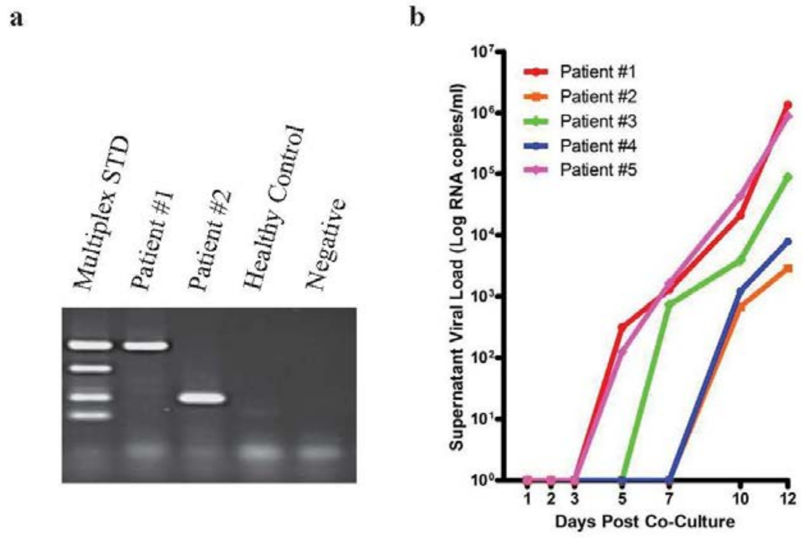

Extended Data Fig. 1. Infectious dengue virus was in the fraction of micro-particles. Acute dengue plasma was collected as described previously [1]. The plasma was subjected to sequential centrifugation at 1,500g for 15 minutes to remove larger particles. The resulted supernatant was further high speed centrifuged at 13,000g for 30 minutes to collected micro-particles. The resulted pellets were resuspended in 100 micro-litter culture media. Five micro-litter was used for dengue viral RNA isolation and nested PCR for serotype identification was done as described previously [1], and ten micro-litter were used for co-culturing with Vero cells. The supernatant of the co-culture was harvested at indicated time and the dengue viral RNA was isolated and subjected to quantitative RT-PCR as described previously [1]. **(a)** Presence of dengue viral RNA in micro-particle. The expected size of the nested PCR

products for DENV1, DENV2, DENV3, and DENV4 were 504 bp, 346 bp, 196 bp, and 143 bp, respectively. As such patient #1 is DENV1 and patient #2 is DENV3. Microparticles from healthy donors were used as a control. Negative control is without primers to perform the reaction. **(b)** Infectious dengue virus in microparticles isolated from acute dengue patients.

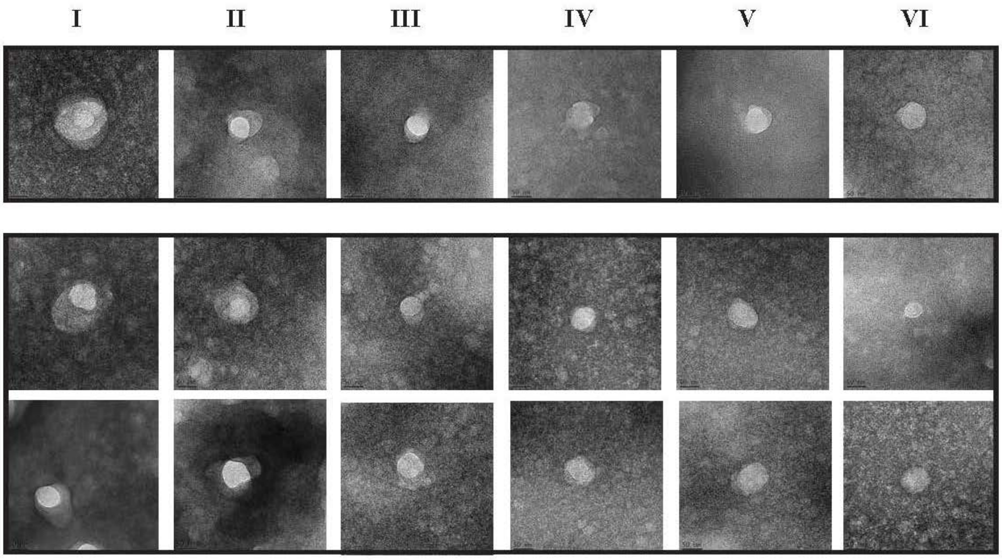

Extended Data Fig. 2. Gallery of the heterogeneous dengue vesicles.

As mentioned in (Fig. 3) Dengue vesicles were organized into six distinct populations (top panel I to VI) by the ratio of the inner particle to the membrane associated. Additional representative figures are presented below each group to show the variation between groups of dengue vesicles.

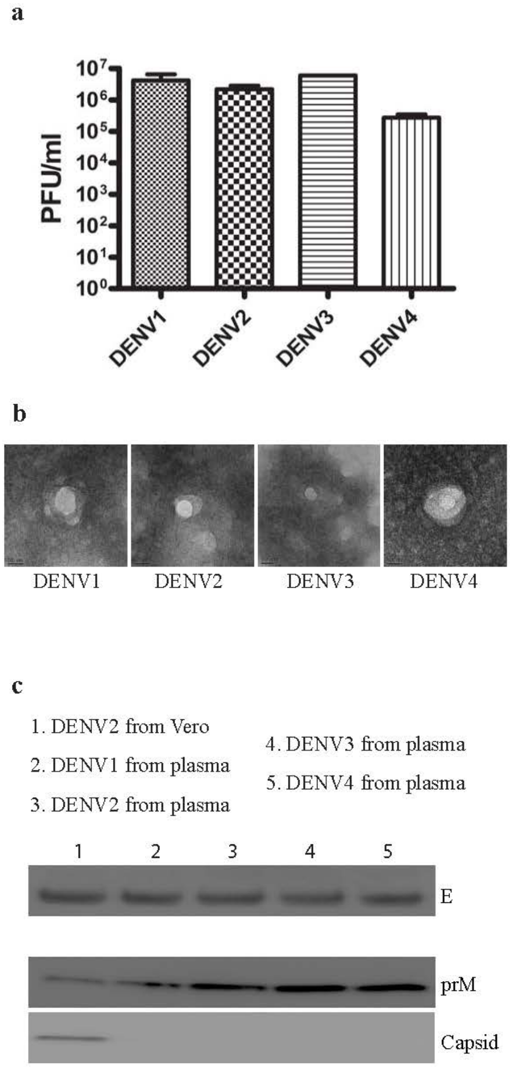

Extended Data Fig. 3. *In vivo* viral properties and morphologies were similar among all four dengue serotypes.

The graph demonstrated the high viral titers used for the capture of EM images for four dengue serotypes from acute dengue plasma **(a)** and showed that the sunny-side up appearance in acute dengue plasma was observed in all serotypes of dengue virus **(b)**. Western blot of dengue envelope, pre-cursor membrane, and capsid protein of all 4 dengue serotypes showed that all serotypes of dengue vesicles did not contain the capsid protein **(c)**. The capsidless and membrane associated morphology is a universal biological property among all dengue vesicles, regardless of serotypes.

a b

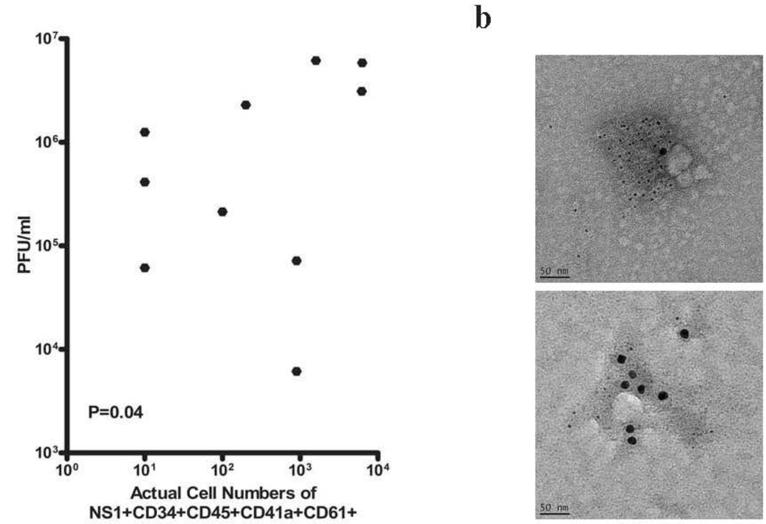

Extended Data Fig. 4. Surface markers with megakaryocytic lineage cells were correlated with viral load and associated with dengue vesicles. (a) Actual cell count numbers based upon FACS analysis for the stem/progenitor of megakaryocytic lineage cells (surface markers of CD34+CD45+ CD61+CD41a+) present in the peripheral blood of acute dengue patients directly correlated to the viral titers in the patient plasma. **(b)** Human CD61 (conjugated with 6nm gold) was present on the membrane of the dengue vesicle along with the dengue envelope protein (conjugated with 18nm gold). The components of the membrane on the dengue vesicles were explored utilizing double staining immuno-EM as described in the Materials and Methods. The results suggested the origin of the dengue vesicle might be of the megakaryocytic lineage.

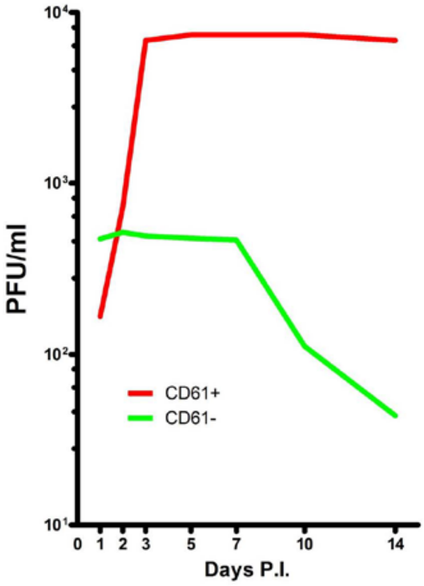

Extended Data Fig. 5. CD61+ cells supported significantly higher DENV replication than CD61- cells in human bone marrow.

Freshly obtained human bone marrow was stained with anti-human CD61 antibody conjugated with magnetic beads to sort out CD61+ cells from the bone marrow. When infected with the same cell number and same MOI by DENV, CD61+ cells gave rise to higher DENV titers compared to CD61- cells. This result suggests that the dominant permissive cells to DENV in human bone marrow are of the megakaryocytic lineage cells which bear the CD61 marker.

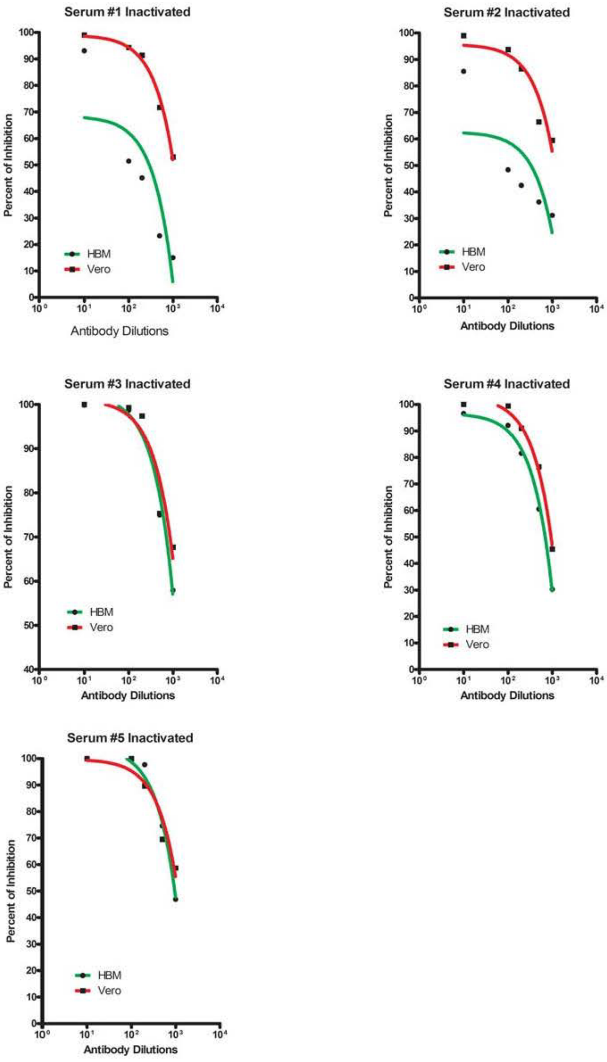

Extended Data Fig. 6. PRNT profiles of dengue vesicles and classical dengue virus with dengue patient convalescent sera. Neutralizing antibody assays were performed as previously described. Percentage of inhibition was calculated by comparing the number of the plaques surviving antibody neutralization with the corresponding serum dilution performed without antibody.

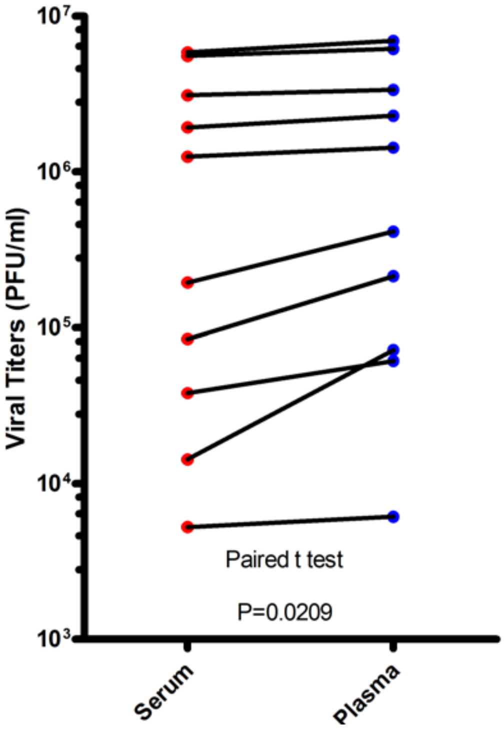

Extended Data Fig. 7. Dengue viral titers in acute plasma were significantly higher than that of serum. Standard plaque assays were performed as mentioned in the Method section. Paired t test revealed that higher viral load was observed in acute dengue plasma than that of acute parallel serum, P=0.0209.

1 Noisakran, S. *et al.* Detection of dengue virus in platelets isolated from dengue patients. *Southeast Asian J Trop Med Public Health* **40**, 253-262 (2009).
